# What characterizes effective tooth brushing of daily users of powered versus manual toothbrushes?

**DOI:** 10.1186/s12903-022-02045-0

**Published:** 2022-01-16

**Authors:** Waldemar Petker-Jung, Ulrike Weik, Jutta Margraf-Stiksrud, Renate Deinzer

**Affiliations:** 1 Department of Medicine, Justus-Liebig-University Giessen, Klinikstr. 29, 35392 Giessen, Germany; 2 Marburg, Germany

**Keywords:** Oral health, Oral hygiene, Tooth brushing, Dental devices, Home care, Powered tooth brush, Dental plaque, Behaviour observation techniques

## Abstract

**Background:**

Recent data show comparable deficits in oral cleanliness after tooth brushing in habitual users of powered toothbrushes (PT) and manual toothbrushes (MT). The present analysis explores the origin of these deficits by relating aspects of the observed tooth brushing behaviour to plaque after tooth brushing.

**Methods:**

Users of rotating-oscillating PT (N = 48) and of MT (N = 52) brushed their teeth the best they could while being filmed. Video analyses assessed brushing time, number of sextants brushed sufficiently long (7.5 s per surface; NSBSL), brushing of outer surfaces with closed jaws, and brushing movements. Correlation analyses examined the relationship between these parameters and plaque after brushing (Marginal Plaque Index (MPI); Turesky modification of Quigley Hein Index (TQHI)) and gingivitis (Papillary Bleeding Index (PBI)).

**Results:**

In PT users, correlations between behaviour and MPI-scores were significant for the NSBSL (outer surfaces: rho = − 0.249; inner surfaces: rho = − 0.510) and brushing duration (outer surfaces: rho = − 0.399; inner surfaces: rho = − 0.509). In MT users, vertical movements on the outer surfaces were positively related to MPI (rho = 0.299). In contrast, circular movements correlated negatively with MPI in those who brushed all outer sextants sufficiently long (n = 47: rho = − 0.294). In both groups, PBI-scores on the inner surfaces were negatively correlated to NSBSL and brushing duration (rho = − 0.327 − rho = − 0.246).

**Conclusion:**

NSBSL and brushing duration appear to play an important role for brushing effectiveness and gingival health in PT and MT users. Whether PT users apply brushing movements or not apparently does not affect the result. In MT users, circular movements seem to be more efficient than vertical movements on the outer surfaces.

**Supplementary Information:**

The online version contains supplementary material available at 10.1186/s12903-022-02045-0.

## Background

The goal of tooth brushing is to remove plaque and thereby to prevent plaque-induced diseases and maintain oral health. Although manual toothbrushes are a well-established device for this, recent studies show that people have difficulties achieving oral cleanliness after manual tooth brushing [1–5]. Despite the instruction to perform oral hygiene to the best of one’s abilities, high levels of plaque persist after tooth brushing, especially adjacent to the gingival margin.

Powered toothbrushes are an alternative to manual toothbrushes. Hence, a recent study analysed whether habitual users of a powered toothbrush (PT) would achieve better results in terms of oral cleanliness after brushing as compared to habitual users of a manual toothbrush (MT) [7]. In this study, no group differences were found with respect to oral cleanliness after participants brushed their teeth to the best of their abilities. Though brushing duration exceeded general recommendations [6], plaque persisted at almost half of the sections of the gingival margin in both groups [7]. This suggests an ineffective brushing performance. Thus, the question arises as to what characterizes the tooth brushing behaviour of both groups and how these aspects relate to brushing effectiveness in terms of oral cleanliness after brushing.

With regard to manual tooth brushing there are already several studies indicating that the effectiveness of brushing increases with an even distribution of brushing time across all surfaces and the time brushed by circular movements [4, 8]. However, to date, no such analysis is available for powered tooth brushing.

The present analysis thus focuses on the brushing behaviour of the participants of the study mentioned above [7] and relates it to the oral cleanliness after brushing. The major aim of the analysis is to find out which aspects of tooth brushing behaviour (overall brushing duration, brushing duration per sextant and surface, brushing movements, brushing of two jaws at a time (tiger bite)) predict the oral cleanliness after tooth brushing in users of powered and manual toothbrushes, respectively?

## Methods

### Ethics

The study protocol was conducted according to the principles of the Declaration of Helsinki and was approved by the local Ethics Committee of the Medical Faculty of the University of Giessen (reference number 124/16). All participants provided informed written consent.

### Participants

Participants were university students from Giessen (Hesse, Germany), who habitually used either a powered toothbrush with a technology of rotating-oscillating movements (PT; N = 48) or a manual toothbrush with vertical bristles arrangement (MT; N = 52). The recruitment was carried out through distribution of posters in the campus, advertisements in local magazines and internal e-mail circulars. A flow diagram illustrating the recruitment process is shown in additional file 1. The inclusion criteria were: (1) 18–30 years of age, (2) at least 20 natural teeth; (3) use of the specific type of toothbrush (PT vs. MT) predominantly (at least 2/3 of all brushing events per week) and habitually (since at least 6 months). Participants were excluded if one of the following criteria applied: (1) study of dentistry/medicine, (2) fixed-orthodontic appliances, (3) removable dentures, (4) physical impairment that affects oral hygiene, (5) dental prophylaxis within the previous four months, (6) pregnancy, and (7) antibiotic therapy within the previous six months. Initially, recruitment was not limited to a specific type of PT or MT. After completion of recruitment, it turned out that the majority of participants used PTs with rotating-oscillating technology or MTs with vertical bristle arrangement. In order to reduce unsystematic variance, the current and the former analyses [7] are confined to these participants and exclude those n = 15 participants who used other toothbrush technologies (e.g. sonic toothbrush) or MTs with angled bristles (see flow diagram provided as Additional file 1).

### General design

This is a cross-sectional study. Interested students were contacted by telephone, informed about the study and screened regarding their eligibility. Eligible students were invited and instructed to bring their own toothbrush and to abstain from any oral hygiene for at least four hours before the appointment. At the beginning of the examination, they received written and oral information about the study procedure and obtained written consent. Then dental status, gingival bleeding and plaque before brushing were assessed. In a separate oral hygiene room, the students were asked to brush their teeth with their own toothbrush to the best of their abilities. They were asked to stand in front of a tablet computer, which served both as a mirror and as a camera. Besides conventional toothpaste, floss and interdental brushes in different sizes were provided. There was no time limit and the time was not displayed on the tablet computer. During oral hygiene performance, the participants were left alone but videos were recorded simultaneously. Immediately after oral hygiene, the remaining plaque was assessed. Video analyses of brushing behaviour started only after the completion of the data collection.

### Clinical assessment

Clinical data were assessed by a calibrated dental examiner (WP) who was blinded to the condition (PT vs. MT) of the respective participant. Details of calibration and the clinical examination are provided in Petker et al. [7]. The present analysis focuses on plaque after tooth brushing to the best of one’s abilities as measured by the Marginal plaque index (MPI) [9], as well as by the Quigley and Hein Index modified by Turesky (TQHI) [10]. The former allows for a detailed assessment of plaque adjacent to the gingival margin, while the latter assesses plaque over the entire surface of the tooth crown in five different degrees. The papillary bleeding index (PBI) by Saxer and Mühlemann [11] as modified by Rateitschak [12] served as a measure of gingivitis and indicator of enduring hygiene deficits.

### Observed oral hygiene behaviour

Video analysis was performed by three independent calibrated observers (WP, and two assistants PK and KB) using the video observation software Mangold Interact 16 (Mangold International GmbH, Arnstorf, Germany). For calibration, five different videos of a former observational study on oral hygiene behaviour with a manual toothbrush were used [8]. Furthermore, two videos of excluded subjects using a powered toothbrush [7] were used for calibration. Calibration procedure was considered successful for interclass correlations (ICC) > 0.90. To ensure the reliability of coding after calibration, double coding was performed for at least 5 videos per observational category by a second observer. The primary observer did not know which videos were double coded by another person and both observers were blinded with respect to the observations of the other one. From a value of ICC > 0.8, it was assumed that the primary observer did not deviate from the previously established standards. ICCs of these observations were all > 0.806.

The observers viewed the brushing videos multiple times in slow motion. During the first run, WP and PK assessed the overall brushing duration (total time of contact of the bristles to the tooth surface). Next, the localization of brushing with respect to the tooth surface (inner, i.e. palatinal/lingual surfaces, outer i.e. buccal/labial surfaces and occlusal surfaces) was assessed. As plaque was only measured on the inner and outer surfaces, the following video analyses were confined to these surfaces. KB analysed for how long each of the sextants (1–6) was brushed. If a participant brushed outer surfaces with the jaws closed (so-called tiger bite) and thereby brushed two antagonistic sextants at a time (sextant 1 plus 6; sextant 3 plus 4; sextant 2 plus 5), this brushing time was added to both sextants (see [13]). These data were used to determine the number of sextants brushed sufficiently long (NSBSL) or had been neglected whilst brushing the inner and outer surfaces, respectively. Brushing duration of less than 1 s within a sextant per surface is considered as a ‘neglect’. A brushing duration of 7.5 s within a sextant per surface was considered sufficient [1, 13]. Finally, the brushing movements were analysed by WP. For manual tooth brushing, the time during which participants applied horizontal, circular or vertical brushing movements or the modified bass technique was recorded. For rotating-oscillating toothbrushes, manufacturers in general recommend holding the PT against the tooth and to move the brush head slowly from tooth to tooth and spend a few seconds on each tooth surface. There is no necessity to perform other brushing movements with the PT because the toothbrush itself moves the bristles to remove plaque. However, video analysis revealed that PT users still applied manual brushing movements. Therefore, the time by which participants applied such movements was assessed.

### Statistics

All statistical analyses were performed using SPSS 27 (IBM). Kolmogorov–Smirnov goodness of fit tests checked for normal distribution. In case of a significant deviation (*p* ≤ 0.05), descriptive statistics are reported as median (Md) and inter-quartile distances (Q3–Q1). In other cases, mean values (M) and standard deviation (SD) are given. To assess which of the behavioural parameters predict oral cleanliness after brushing correlational analyses were run. Spearman rank correlations (rho) were computed instead of Pearson correlations in order to compensate for outlying data and non-normality of the variable distributions. The primary criterion variable was the MPI after brushing; the TQHI was analysed as secondary criterion as it has been shown to be less sensitive to alterations in brushing behaviour than the MPI [8]. As a third criterion the mean percentage of TQHI values above 2 (%TQHI 3–5) was included. TQHI values of 1 and 2 reflect plaque confined to the gingival margin and thus depict what is already measured by the MPI. TQHI values scoring 3–5 indicate plaque that is not confined to the gingival margin but extends to the more coronal parts of the teeth. Papillary bleeding (PBI mean score and percentage of sites bleeding) was the fourth criterion. Predictor variables were the following behavioural parameters: brushing duration of lateral surfaces, percentage of time manual brushing movements were applied at lateral surfaces (only for users of a powered toothbrush), percentage of time specific brushing movements like circular, horizontal and vertical movements were applied at lateral surfaces (only for users of a manual toothbrush), NSBSL on the outer and at inner surfaces, the number of sextants neglected (brushing duration of less than 1 s), percentage of time by which outer surfaces were brushed with the jaws closed (tiger bite), respectively. Significance level was set at α = 0.10. This would allow for the detection of medium effect sizes (rho = 0.35) with a power of 1 − β = 80%.

Mann Whitney U-tests were run to see whether groups differed with respect to %TQHI 3–5, number of sextants neglected, number of sextants brushed sufficiently long and percentage of time they applied the tiger bite.

## Results

Characteristics of the sample and the distribution of brushing time to surfaces have been described earlier [7]. Briefly, the sample comprised N = 48 PT users and N = 52 MT users (71 female / 29 male) with an average age of 24.6 ± 3.2 years. Most of them were non-smokers (5 smokers). Mean number of teeth was 28.6 (min = 24; max = 32). Mean PBI was 0.17 (± 0.12), PBI % was 9.32 (Q1 = 5.36, Q3 = 14.46) and most of the participants (N = 96) showed no clinically detectable caries lesions. MPI values (% of sections with plaque) after brushing were close to 40% in both groups with no significant group difference (*p* = 0.80) and mean TQHI values after brushing were TQHI = 1.2 in both groups (*p* = 0.95). Total brushing duration exceeded 3 min in both groups with no significant group difference (*p* = 0.18). In addition, no group differences were found with respect to duration of brushing occlusal (*p* = 0.07) or outer surfaces (*p* = 0.26) but PT users brushed their inner surfaces significantly longer (*p* = 0.01) than MT users (see [6]). The current analyses also revealed no difference with regard to percentage of TQHI values exceeding TQHI = 2 (%TQHI 3–5) on the inner or outer surfaces after oral hygiene (*p* > 0.72). These analyses also revealed extremely low %TQHI 3–5 values on the inner surfaces in most participants (see Additional file 2).

### Distribution of brushing time to sextants

**Fig. 1:**
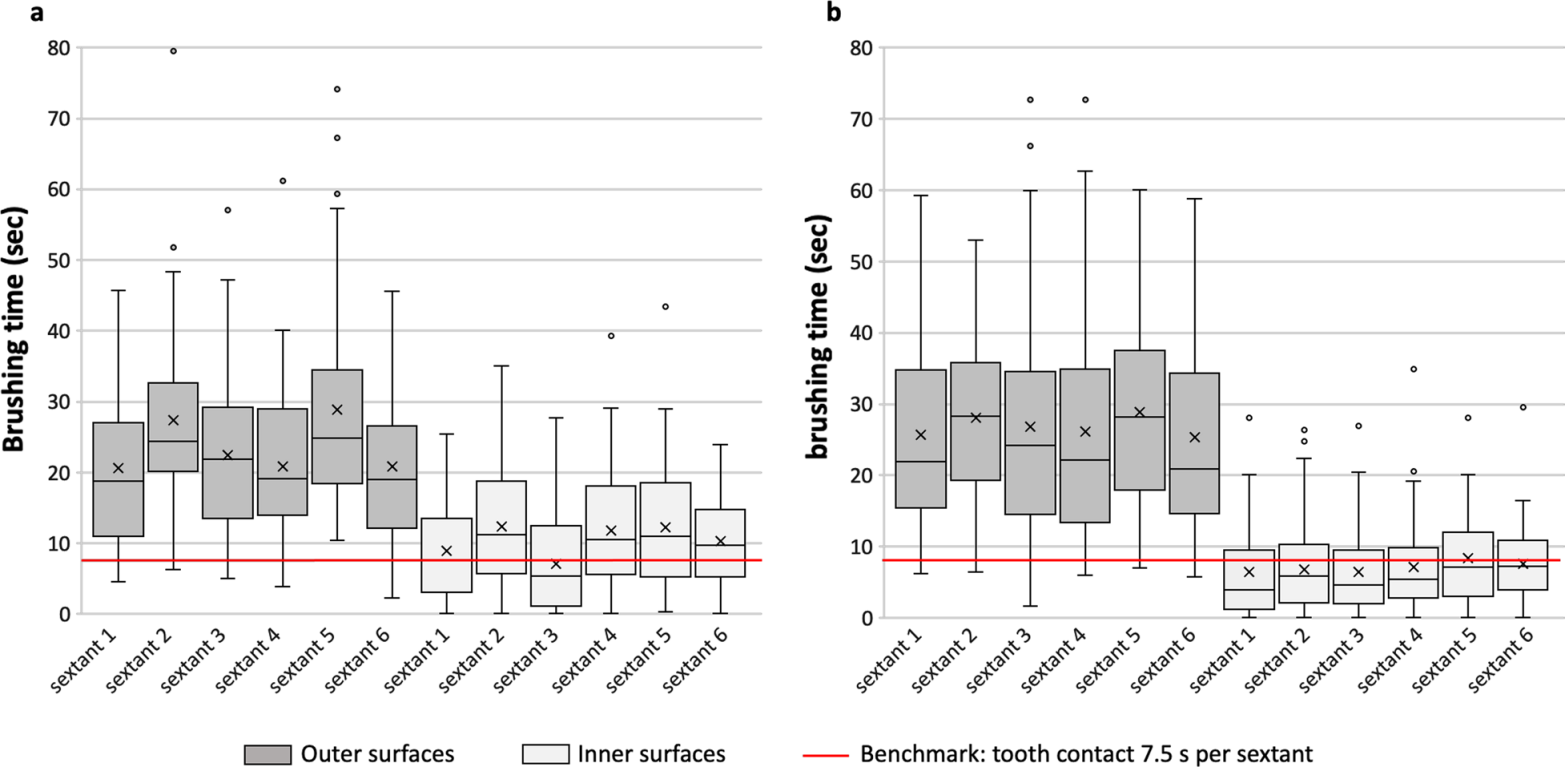
Distribution of brushing time of sextants on the outer and inner surfaces for PT (**A**) and MT (**B**) users, respectively. The line in the middle of the box represents the median, the upper and lower borders the 25% (Q1) and 75% (Q3) quartiles respectively. The whiskers represent the highest/lowest value that is still within the limits for outlier values (1.5 times the interquartile distance) and the dots show extreme values. The red line marks a time of 7.5 s per surface sextant, which is considered sufficiently long

Figure 1 shows the distribution of brushing time for PT (A) and MT users (B) on the inner and outer sextant surfaces, respectively. Since some values deviate from the normal distribution, all values are presented as boxplots. Nearly all PT users (n = 42) and all MT users applied the tiger bite while brushing the outer surfaces at least to some extent (percentage of brushing time: PT: Md = 29.26, Q1 = 9.04, Q3 = 73.88; MT: Md = 86.36, Q1 = 59.93, Q3 = 96.79; *p* < 0.001). Groups did not differ significantly regarding the number of outer sextants they brushed sufficiently long (exact *p* = 0.122). Most participants of both groups managed to brush all outer sextants by at least 7.5 s (NSBSL = 6; n = 38 PT users and n = 47 MT users). Regarding inner sextants PT users brushed more sextants sufficiently long than MT users (PT: Md = 3.5 NSBSL, Q1 = 2, Q3 = 5.7; MT: Md = 2 NSBSL, Q1 = 0, Q3 = 4; exact *p* = 0.005), however 33% of PT users and 37% of MT users neglected at least one sextant (see Additional file 3). No group differences were found with regard to the number of sextants neglected (*p* = 0.57).

### Brushing movements

**Fig. 2:**
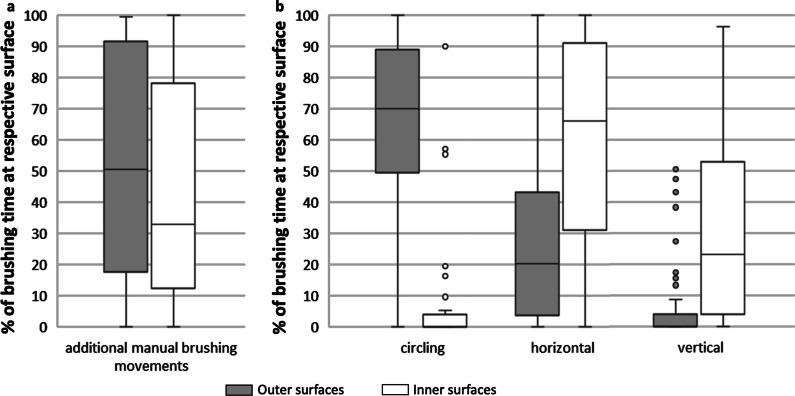
Percentage of time relative to total brushing duration on the outer and inner surfaces, respectively, spent with additional manual brushing movements in PT users (**A**) and specific brushing movements (circular, horizontal and vertical) in MT users (**B**). The line in the middle of the box represents the median, the upper and lower borders the 25% (Q1) and 75% (Q3) quartiles respectively. The whiskers represent the highest/lowest value that is still within the limits for outlier values (1.5 times the interquartile distance) and the dots show extreme values

Figure 2 shows the distribution of brushing movements to inner and outer surfaces. The majority of the powered toothbrush users applied manual brushing movements both on the inner (Md = 32.98% of brushing time; Q1 = 12.37%; Q3 = 78.11%) and the outer surfaces (Md = 50.45% of brushing time; Q1 = 17.51%; Q3 = 91.60%). In users of manual toothbrushes, circular movements predominated on the outer surfaces (Md = 70.15% of brushing time; Q1 = 49.61%; Q3 = 89.07%) and horizontal movements on the inner surfaces (Md = 63.4% of brushing time; Q1 = 28.23%; Q3 = 88.7%). 73% of the participants never showed circular movements on the inner surfaces and 67.3% never showed vertical movements on the outer surfaces. Only two subjects used the modified bass technique (not shown in Fig. 2).

### Prediction of oral cleanliness and gingival bleeding by brushing behaviour

Intercorrelations within predictor variables and within plaque and bleeding data are presented in the Additional file 4. On the inner surfaces %TQHI 3–5 values were extremely low (see Additional file 2) and affected in the majority of participants only three teeth or less. Thus, correlational analyses would have been meaningless and were omitted.

Table 1 shows the results of correlation analyses for the other parameters within PT users. Significant negative correlations between toothbrushing behaviour and plaque were found for brushing time and the NSBSL both on the outer and inner surfaces. PBI-scores on the inner surfaces were also negatively correlated to the NSBSL and brushing duration. The percentage of applying the tiger bite correlated positively with % THQI 3–5 and on the outer surfaces, the number of neglected sextants was positively related to plaque scores on the inner surfaces.

**Table 1:** Prediction (Spearman rho) of plaque after brushing and gingival bleeding by behavioural parameters in PT users (n = 48)

	Brushing time	Brushing movement (% of brushing time)	NSBSL (brushed ≥ 7.5 s)	Number of neglected sextants (brushed ≤ 1 s)	Tiger bite* (% of brushing time)
Outer surfaces					
MPI	**−.399**	−.224	**−.249**	b	.152
TQHI	**−.276**	−.181	−.117	b	.184
% TQHI 3–5	**−.398**	−.121	−.171	b	**.288**
PBI	.159	−.107	.180	b	.169
% PBI	.162	−.131	.160	b	.151
Inner surfaces					
MPI	**−.509**	−.101	**−.510**	**.422**	–
TQHI	**−.405**	−.096	**−.412**	**.345**	–
% TQHI 3–5	a	a	a	a	–
PBI	**−.246**	−.053	**−.293**	.220	–
% PBI	**−.276**	−.030	**−.300**	.212	–

Bold figures indicate significant values. a: not enough variance in this parameter for meaningful correlations (% TQHI 3-5 values were extremely low on the inner surfaces); b: no variance in this parameter (all participants brushed all sextants at least 1 s); *not applicable on inner surfaces

Table 2 shows how behavioural parameters relate to plaque after brushing and gingival bleeding in MT users.

**Table 2:** Prediction (Spearman rho) of plaque after brushing and gingival bleeding by behavioural parameters in MT users (n = 52)

	Brushing time	Brushing movement (% of brushing time)^#^			NSBSL (brushed ≥ 7.5 s)	Number of neglected sextants (brushed ≤ 1 s)	Tiger bite* (% of brushing time)
		Circular	Horizontal	Vertical			
Outer surfaces							
MPI	−.186	−.174	.006	**.299**	a	b	−.179
TQHI	−.095	−.182	.034	.221	a	b	−.026
% TQHI 3–5	−.209	−.130	−.036	.093	a	b	.020
PBI	−.162	−.177	.004	.199	a	b	−.133
% PBI	−.113	−.180	−.021	.161	a	b	−.125
Inner surfaces							
MPI	−.161	.129	.110	−.143	−.199	.004	–
TQHI	.003	.108	.104	−.064	−.064	−.096	–
% TQHI 3–5	a	a	a	a	a	a	–
PBI	**−.287**	.023	.017	−.126	**−.308**	−.045	–
% PBI	**−.275**	.073	.053	−.161	**−.327**	−.083	–

Bold figures indicate significant values. a: not enough variance in this parameter for meaningful correlations (n =  47 out of n = 52 participants brushed all sextants ≥ 7.5 s; % TQHI 3-5 values were extremely low on the inner surfaces); b: no variance in this parameter (all participants brushed all sextants at least 1 s); *not applicable on inner surfaces; #n = 49, as correlations for inner surfaces do not include 3 participants who did not brush inner surfaces at all.

In MT users, vertical movements on the outer surfaces were positively related to MPI (rho = 0.299). On the inner surfaces, no significant correlations between behaviour and plaque were found. PBI-scores on the inner surfaces were negatively correlated to the NSBSL and brushing duration (rho = − 0.327 − rho = − 0.275). Since only 5 MT users did not brush outer surfaces sufficiently long, additional correlational analyses with the remaining participants (n = 47) were calculated to explore the relationship between the other behavioural parameters and plaque. In these analyses the percentage of circular movements significantly predicted marginal plaque after brushing (rho = − 294, *p* = 0.045) and TQHI mean values (rho = − 0.330, *p* = 0.023) but not %TQHI3-5 values (rho = − 0.165, *p* = 0.267). Vertical strokes remained to be positively related to marginal plaque after brushing within these participants (rho = 0.292, *p* = 0.047).

## Discussion

Video observations revealed remarkable similarities in the brushing behaviour of habitual PT users and MT users. Both groups showed a similar brushing duration and did not neglect any outer sextants while brushing. Instead, in both groups, most participants (> 79%) brushed all outer sextants sufficiently long. The groups do differ significantly in the extent they neglected the inner surfaces. Nevertheless, similar to the MT users in the current and previous analyses [1, 5, 14], the PT users brushed their inner surfaces shorter and brushed fewer inner sextants sufficiently long compared to outer sextants. A similar pattern regarding distribution of brushing time across surfaces and neglect of inner sextants was found in another study [15] instructing PT and MT users to brush their teeth as usual. This indicates that irrespective of the instruction (brushing as usual or brushing to the best of one’s abilities) PT users tend to neglect brushing their inner surfaces just as MT users do. Similarities in behaviours are also visible in aspects where one would rather not expect this. In Germany, a common advice children receive for brushing their outer surfaces is to brush them with circular movements, two antagonistic sextants at a time, while slightly closing their jaws (brushing in the tiger bite [13]). For the usage of a PT, no such advice is known. Nevertheless, most of the PT users applied the tiger bite, though less extensively. PT users also applied brushing movements for a considerable proportion of time despite the absence of a recommendation to this effect. According to suggestions of the manufacturer of the most popular oscillating rotating PT system, one would just move the toothbrush slowly from tooth to tooth [16]. One might assume that in the current study PT users wanted to make brushing more effective by moving the brush because they were asked to clean their teeth as good as possible. Still, a similar pattern was observed in PT users when they were instructed to brush as usual [15]. Summarizing these behavioural analyses, brushing behaviour seems to only moderately depend on the type of toothbrush used.

The major aim of the current study was to find out which of the behavioural aspects relate to oral cleanliness after brushing. For PT users no such data were available so far. The analyses revealed that brushing time in PT users was the strongest predictor of oral cleanliness after brushing. NSBSL and the number of sextants neglected was also related to plaque after brushing. Manual movements while brushing with PT seem meaningless, although the direction of the results suggests that they might be beneficial rather than harmful. The opposite seems to be true for the tiger bite. This parameter turned out to be positively related to plaque after brushing which became significant for the more coronal parts of the plaque, indicating that applying the tiger bite when brushing with PT seems to be an ineffective behaviour regarding brushing success.

With respect to MT users correlational analysis between plaque scores and behavioural parameters are less explicit. Regarding inner surfaces, no significant correlations were found, even though the direction of results and the correlations to gingival bleeding point in the same direction found in PT users: NSBSL and the overall brushing time were most strongly related to brushing success and gingival bleeding. The less pronounced correlations might be due to differences in the distribution of brushing time of inner surfaces. Figure 1 shows considerably larger interindividual variance with respect to this parameter in PT users as compared to MT users. Thus, the lower correlation coefficients might be a statistical artefact due to a lower behavioural variance within this group. With respect to outer surfaces, vertical movements were positively related to gingival plaque in MT users. An additional analysis within the subgroup of MT users who brushed all outer sextants sufficiently long showed that circular brushing movements best predicted oral cleanliness after brushing. Considering these analyses, circular movements might be superior to vertical movements when it comes to the oral cleanliness in MT users. These data correspond to what has been observed in two other observational studies which also found circular movements to be predictive for plaque levels [4, 8]. Additionally, experimental studies found circular brushing movements to be of superior efficacy as compared to vertical movements [3, 17].

Since it is unclear to what extent the plaque values achieved under laboratory conditions reflect oral hygiene at home, gingival inflammation was also assessed as a measure of long-term oral cleanliness. In both groups, gingival health is predicted by brushing time and NSBSL on the inner surfaces. The brushing behaviour shown by the study participants in the laboratory therefore seems to reflect, at least partly, the daily brushing behaviour. However, this association is not evident on the outer surfaces. Since most subjects brushed outer surfaces longer and more completely, this might be again due to less variance in brushing parameters on the outer surfaces.

Even though PT and MT users did not differ with respect to plaque levels after oral hygiene, these could be more readily predicted by brushing behaviour in PT as compared to MT users. As discussed above, limited variance within some parameters might be one explanation. Another refers to the method of video analysis. Even though the observers and methods were the same both for PT and MT users, there are some important differences between the two groups which might affect the reliability of the observation and thereby variance explanation: First, the brush heads of manual toothbrushes are sized larger than the ones of rotating-oscillating toothbrushes, which makes it more difficult to evaluate the precise location of bristles in relation to surfaces. Second, the cleaning movements of the bristles are an inbuilt factor in the PTs observed here. The oscillating rotating technology standardises them to a high degree. In MT users the cleaning movements of the bristles are under their control and even within a category like circular movements there is a high variance regarding the amplitude and exact form of the movement. A circular movement might be more or less ellipsoid and covers one or more teeth within one movement. This contributes considerably to the error variance of the predictor and thus might lead to a greater proportion of unexplained variance in the criterion. This leads to the limitations of the present study.

The limitations include the accuracy of video analysis already mentioned above (limited evaluation of the precise location of bristles in relation to the gingival margins and differentiation of brushing movements). Furthermore, one should consider that the present study reflects behaviour shown under laboratory conditions when people are instructed to brush their teeth to the best of their abilities. Other behaviours and other relationships between behaviour and plaque might be found under conditions of brushing at home. Nevertheless, gingival inflammation indicated at least some correlation between brushing at home and in the lab. Finally, correlational data presented here should be interpreted with caution. They do not justify causal inferences and rather give hints of the meaningful parameters for plaque removal.

## Conclusion

The present analysis extends the insights of an earlier analysis of these participants [7]. The first analysis had already shown that PT and MT users did not differ in their ability to achieve oral cleanliness. The current analysis also reveals remarkable similarities in their brushing behaviour. Both PT and MT users neglect their inner surfaces. This underlines that the use of a PT does not automatically result in more comprehensive tooth brushing. Furthermore, PT users apply manual brushing movements and the tiger bite like MT users do, even though this seems to be little appropriate for PT with oscillation rotation technology. Within PT users, the duration and comprehensiveness of brushing appears to be the most meaningful predictor of oral cleanliness while in MT users the kind of brushing movements they apply appears to be relevant as well. Still, both groups had difficulties to remove plaque especially at the gingival margins. It thus appears to be important to direct the users’ attention to this zone. Overall, the results of the current study underscore the importance of analysing the tooth brushing behaviour in detail in order to better understand deficits. They also highlight the need for future research specifically addressing methods to overcome behavioural problems observed in PT and MT users.

## Supplementary Information

**Additional file 1**. Flow diagram of participant recruitment.

**Additional file 2**. Percentage of TQHI values higher than 2 for MT and PT users on the outer and inner surfaces, respectively. The line in the middle of the box represents the median, the upper and lower borders the 25% and 75% quartiles respectively. The whiskers represent the highest/lowest value that is still within the limits for outlier values (1.5 times the interquartile distance) and the dots show extreme values.

**Additional file 3**. Number of sextants by sites brushed sufficiently long (≥ 7.5 s) and neglected (≤ 1 s) for the outer and inner surfaces for PT (**A**) and MT (**B**) users.

**Additional file 4**. Intercorrelations (rho) among predictor variables in PT users (N = 48) and MT users (N = 52), respectively.

## Data Availability

The datasets used and/or analysed during the current study are available from the corresponding author on reasonable request. For privacy reasons, however, individual data allowing for the identification of participants (e.g. videos) cannot be made available.

## Abbreviations

ICC: Intraclass correlation
KB: Katarzyna Bandurka (assistant during analyses)
MPI: Marginal Plaque Index
MT: Manual toothbrush
PK: Pascal Kellner (assistant during analyses)
PT: Powered toothbrush
TQHI: Turesky modification of the Quigley and Hein Index
WP: Waldemar Petker (author
