# Supplementary material for: What characterizes effective tooth brushing of daily users of powered versus manual toothbrushes?
Source: BMC Oral Health. 2022 Jan 16;22:10. doi: 10.1186/s12903-022-02045-0 (PMC8762860; doi:10.1186/s12903-022-02045-0)
Supplement: Supplementary file 1 — Additional file 1. Flow diagram of participant recruitment. [file 12903_2022_2045_MOESM1_ESM.pdf]

Flow diagram based on the diagram from Petker et al. (2019)

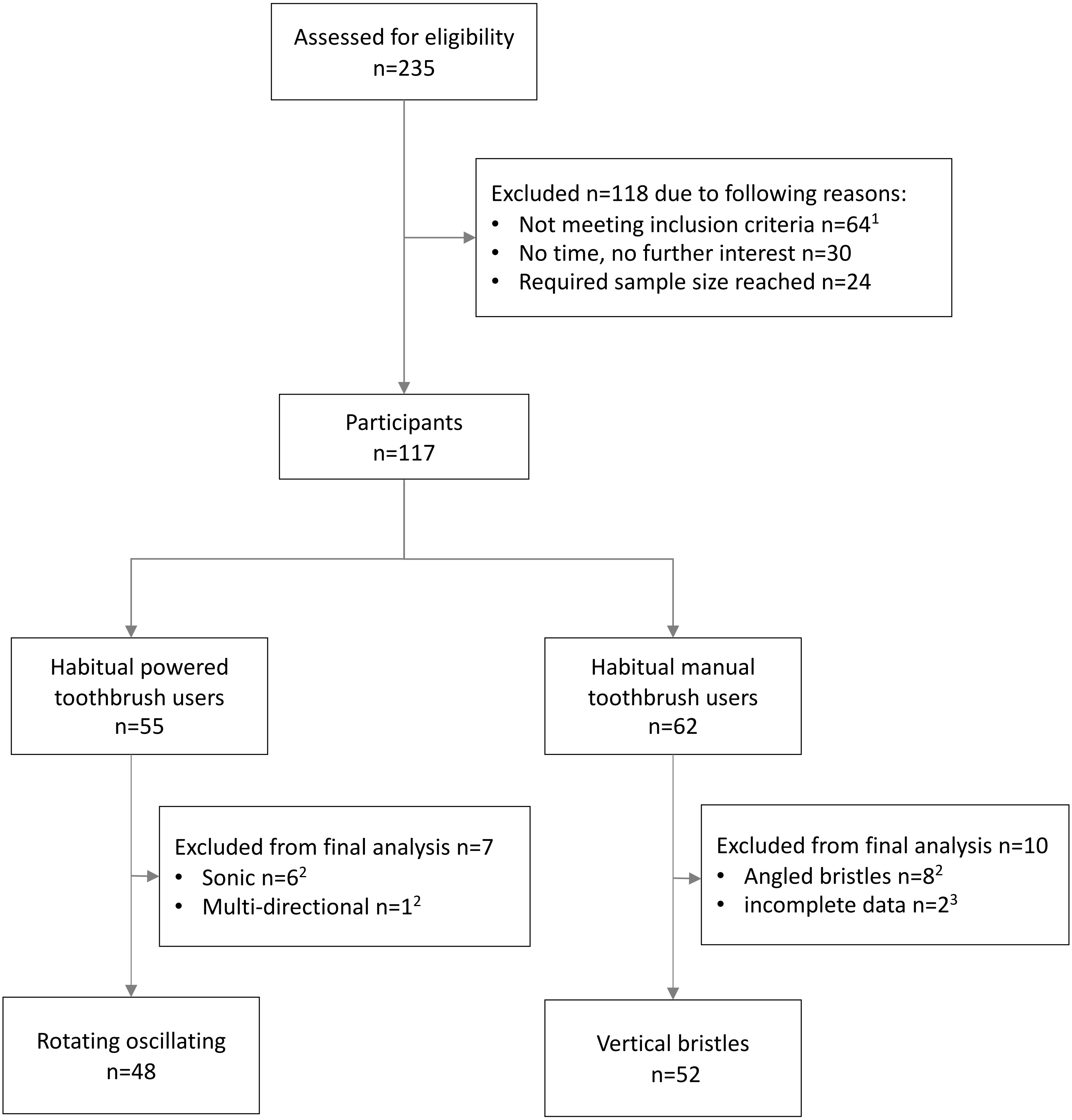

---

[1] For detailed information see flow diagram in Petker et al. (2019).

[2] Participants were excluded in order to reduce unsystematic variance.

[3] Investigation had to be interrupted due to technical difficulties.
