# Supplementary figures and images for: What characterizes effective tooth brushing of daily users of powered versus manual toothbrushes?
Source: BMC Oral Health. 2022 Jan 16;22:10. doi: 10.1186/s12903-022-02045-0 (PMC8762860; doi:10.1186/s12903-022-02045-0)

## Slide 1
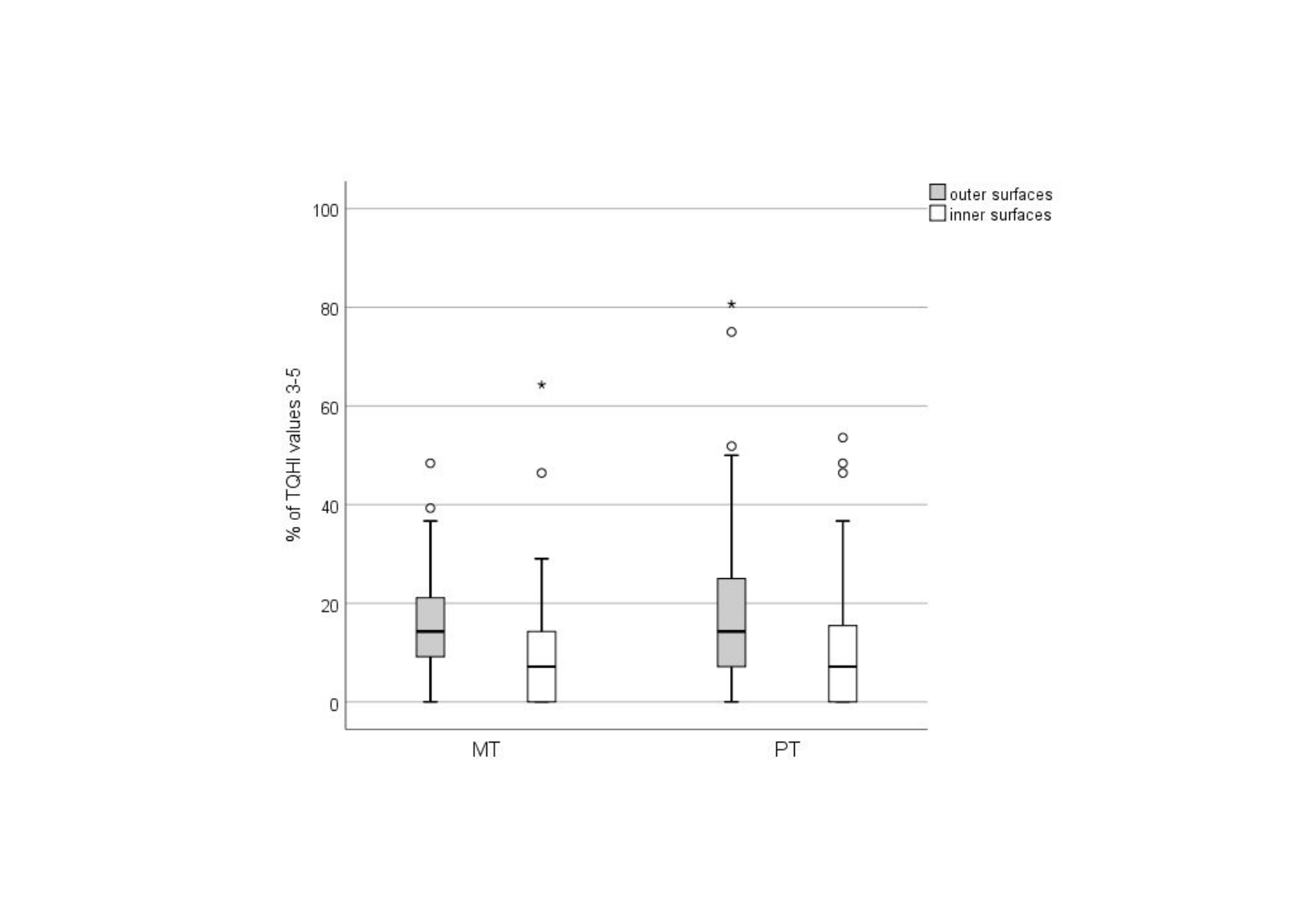

Supplement: Supplementary file 2 — Additional file 2. Percentage of TQHI values higher than 2 for MT and PT users on the outer and inner surfaces, respectively. The line in the middle of the box represents the median, the upper and lower borders the 25% and 75% quartiles respectively. The whiskers represent the highest/lowest value that is still within the limits for outlier values (1.5 times the interquartile distance) and the dots show extreme values. [file 12903_2022_2045_MOESM2_ESM.pptx]

## Slide 1
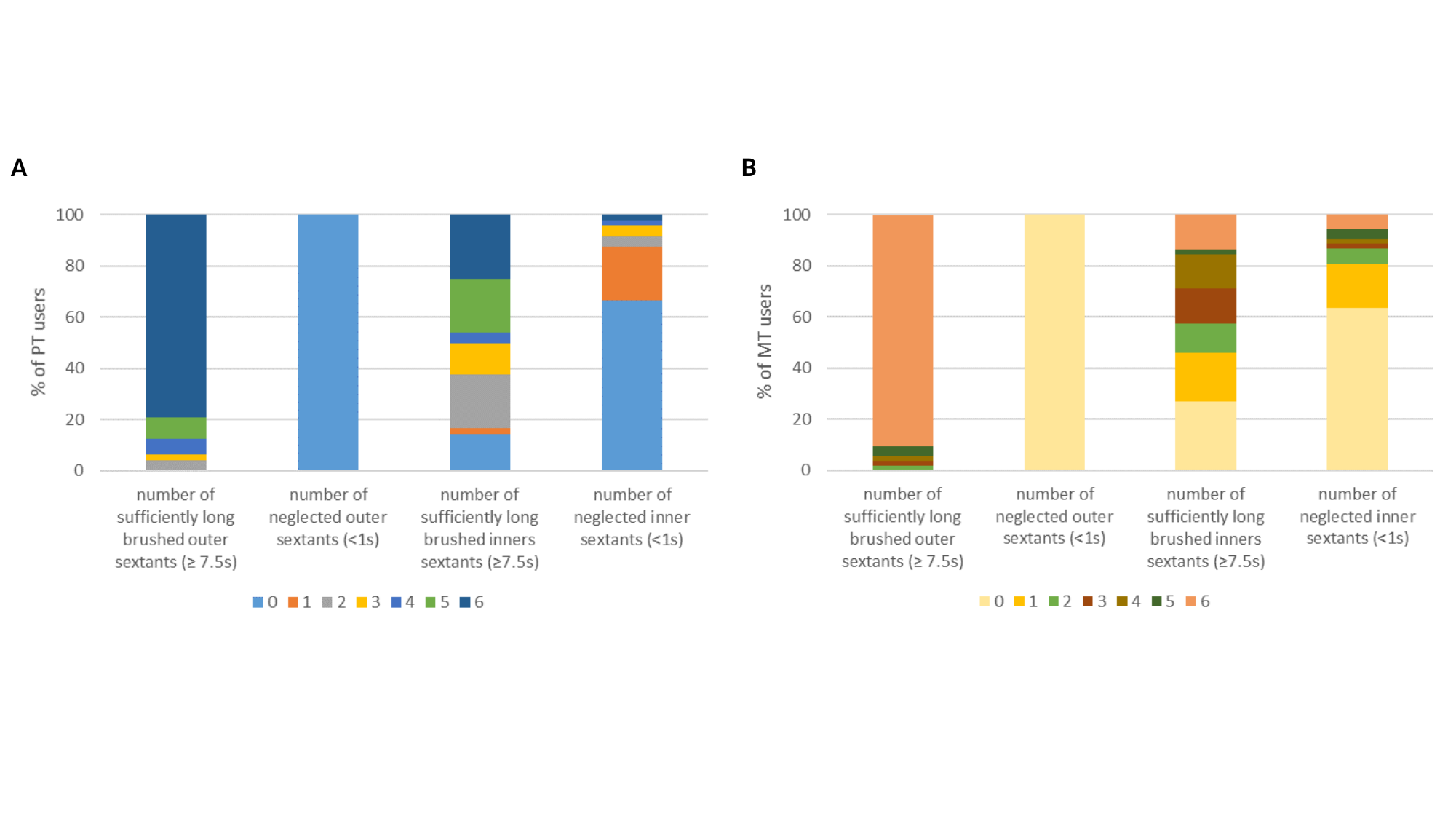

A
B

Supplement: Supplementary file 3 — Additional file 3. Number of sextants by sites brushed sufficiently long (≥ 7.5 s) and neglected (≤ 1 s) for the outer and inner surfaces for PT (A) and MT (B) users. [file 12903_2022_2045_MOESM3_ESM.pptx]
