# Supplementary material for: What characterizes effective tooth brushing of daily users of powered versus manual toothbrushes?
Source: BMC Oral Health. 2022 Jan 16;22:10. doi: 10.1186/s12903-022-02045-0 (PMC8762860; doi:10.1186/s12903-022-02045-0)
Supplement: Supplementary file 4 — Additional file 4. Intercorrelations (rho) among predictor variables in PT users (N = 48) and MT users (N = 52), respectively. [file 12903_2022_2045_MOESM4_ESM.docx]

**Intercorrelations of behavioral parameters and plaque and bleeding indices in PT-users**

|  | Brushing time | Brushing movements  (% of brushing time) | NSBSL (brushed ≥7.5s) | Neglected sextants (brushed <1s) | Tiger bite* (%) | MPI | TQHI | TQHI  3-5 (%) | PBI (mean) | PBI (%) |
| --- | --- | --- | --- | --- | --- | --- | --- | --- | --- | --- |
| Brushing time (s) |  | .077 | .930 | -.644 | -- | -.509 | -.405 | a | -.246 | -.276 |
| Brushing movements (%) | -.120 |  | .193 | -.226 | -- | -.101 | -.096 | a | -.053 | -.030 |
| NSBSL (brushed ≥7.5s) | .607 | -.013 |  | -.643 | -- | -.510 | -.412 | a | -.293 | -.300 |
| Neglected sextants (brushed <1s) | b | b | b |  | -- | .422 | .345 | a | .220 | .212 |
| Tiger bite* (%) | -.270 | .364 | .227 | b |  | -- | -- | -- | -- | -- |
| MPI | -.399 | -.224 | -.249 | b | .152 |  | .957 | .585 | .552 | .585 |
| TQHI | -.417 | -.196 | -.231 | b | .179 | .975 |  | .717 | .530 | .578 |
| TQHI 3-5 (%) | -.398 | -.121 | -.171 | b | .288 | .811 | .869 |  | .448 | .481 |
| PBI (mean) | .159 | -.107 | .180 | b | .169 | .264 | .233 | .131 |  | .972 |
| PBI (%) | .162 | -.131 | .160 | b | .151 | .234 | .194 | .107 | .963 |  |
| Lower triangle: intercorrelations of behavioral parameters and plaque indices at outer surfaces; upper triangle: intercorrelations of behavioral paramters and plaque indices at inner surfaces; a: not enough variance in this parameter for meaningful correlations (% TQHI 3-5 values were extremely low on the inner surfaces); b: at outer surfaces none of the study participants brushed any sextants < 1s; *not applicable at inner surfaces.  Circular/horizontal/vertical (%): Percentage of brushing time spent with circular, horizontal or vertical movements, respectively; NSBSL (brushed ≥ 7.5s): number of sextants brushed at least for 7.5 seconds; Neglected sextants (brushed < 1s): number of sextants brushed less than 1 second; Tiger bite (%): Percentage of brushing time at outer surfaces spent with brushing with mandibular closed; MPI: marginal plaque index; TQHI 3-5 (%): Percentage of sites with TQHI values ≥ 3; PBI (%): Percentage of sites showing bleeding. | | | | | | | | | | |

**Intercorrelations of behavioral parameters and plaque and bleeding indices in MT-users**

|  | Brushing time (s) |  | Brushing movements (% of brushing time)^#^ |  | NSBSL (brushed ≥7.5s) | Neglected sextants (brushed <1s) | Tiger bite* (%) | MPI | TQHI | TQHI  3-5 (%) | PBI (mean) | PBI (%) |
| --- | --- | --- | --- | --- | --- | --- | --- | --- | --- | --- | --- | --- |
|  |  | Circular | Horizontal | Vertical |  |  |  |  |  |  |  |  |
| Brushing time (s) |  | .034 | -.115 | .179 | .910 | -.635 | -- | -.161 | .003 | .320 | -.287 | -.275 |
| Circular (%)^#^ | -.014 |  | -.324 | -.008 | .007 | -.096 | -- | .129 | .108 | .024 | .023 | .073 |
| Horizontal (%)^#^ | -.113 | -.754 |  | -.827 | -.194 | .078 | -- | .110 | .104 | -.046 | .017 | .053 |
| Vertical (%)^#^ | .309 | -.316 | -.097 |  | .211 | -.136 | -- | -.143 | -.064 | .115 | -.126 | -.161 |
| NSBSL (brushed ≥7.5s) | .351 | -.084 | .194 | -.093 |  | -.396 | -- | -.199 | -.064 | .247 | -.308 | -.327 |
| Neglected sextants (brushed <1s) | b | b | b | b | b |  | -- | .004 | -.096 | -.297 | -.045 | -.083 |
| Tiger bite* (%) | .010 | .125 | .018 | -.193 | a | b |  | -- | -- | -- | -- | -- |
| MPI | -.186 | -.174 | .006 | .299 | a | b | -.179 |  | .929 | .207 | .177 | .211 |
| TQHI | -.180 | -.220 | .033 | .234 | a | b | -.176 | .953 |  | .433 | .149 | .147 |
| TQHI 3-5 (%) | -.209 | -.130 | -.036 | .093 | a | b | .020 | .663 | .774 |  | .117 | .024 |
| PBI (mean) | .-162 | -.177 | .004 | .199 | a | b | -.133 | .533 | .543 | .437 |  | .948 |
| PBI (%) | -.113 | -.180 | -.021 | .161 | a | b | -.125 | .494 | .477 | .359 | .950 |  |
| Lower triangle: intercorrelations of behavioral parameters and plaque indices at outer surfaces; upper triangle: intercorrelations of behavioral paramters and plaque indices at inner surfaces; a: not enough variance in this parameter for meaningful correlations (% TQHI 3-5 values were extremely low on the inner surfaces); b: at outer surfaces none of the study participants brushed any sextants < 1s; *not applicable at inner surfaces; ^#^n=49, as intercorrelations for inner surfaces do not include 3 participants who did not brush inner surfaces at all.  Circular/horizontal/vertical (%): Percentage of brushing time spent with circular, horizontal or vertical movements, respectively; NSBSL (brushed ≥ 7.5s): number of sextants brushed at least for 7.5 seconds; Neglected sextants(brushed < 1s): number of sextants brushed less than 1 second; Tiger bite (%): Percentage of brushing time at outer surfaces spent with brushing with mandibular closed; MPI: marginal plaque index; TQHI 3-5 (%): Percentage of sites with TQHI values ≥ 3; PBI (%): Percentage of sites showing bleeding. | | | | | | | | | | | | |
